# IVF success rates in individuals accessing preimplantation genetic testing for monogenic conditions (PGT-M): a single centre retrospective cohort study of 572 IVF cycles

**DOI:** 10.1007/s10815-025-03416-6

**Published:** 2025-03-11

**Authors:** Alice Poulton, Melody Menezes, Tristan Hardy, Sharon Lewis, Lisa Hui

**Affiliations:** 1 Monash IVF Group LTD, Level 1, 510 Church Street, Clayton, Cremorne, VIC 3121 Australia; 2 University of Melbourne, Parkville, VIC Australia; 3 Murdoch Children’s Research Institute, Parkville, VIC Australia; 4 Victorian Clinical Genetics Service, Parkville, VIC Australia; 5 Mercy Hospital for Women, Heidelberg, VIC Australia; 6 The Northern Hospital, Epping, VIC Australia

**Keywords:** Preimplantation genetic testing, Karyomapping, Single gene disorder, Monogenic disorder, 24-chromosome aneuploidy screening, PGT-M

## Abstract

**Purpose:**

To evaluate live birth rates per embryo transfer where the primary indication for assisted reproduction was preimplantation genetic testing for monogenic conditions.

**Methods:**

All oocytes were fertilized using intracytoplasmic sperm injection. On days 5–7, ~ 5 trophectoderm cells were biopsied. Whole genome amplification was performed on biopsy samples, followed by a karyomapping protocol. Embryos underwent concurrent 24-chromosome screening. Outcomes included the number of stimulated cycles resulting in embryo biopsy, monogenic and aneuploidy screening results, embryo transfers, and clinical pregnancies and live births. Generalized Estimating Equations were used to analyze the relationship between binary clinical outcomes and fertility covariates.

**Results:**

Between 2015 and 2022, the laboratory biopsied and tested 2344 embryos for monogenic indications, from 527 stimulated cycles. Eight hundred forty-nine biopsied embryos were euploid and low probability of the condition of interest. Five hundred and thirteen embryos were transferred, resulting in 263 clinical pregnancies, and 230 live births. This translated to clinical pregnancy and live birth rates per embryo transfer of 51.3% (95% CI, 47.0–55.6%) and 44.8% (95% CI, 40.6–49.2%).

Compared with patients undergoing preimplantation genetic testing without a subfertility factor, patients with a subfertility factor were 48% less likely to achieve a clinical pregnancy per embryo transfer (*β* = − 0.4797474, *p* = 0.026) and 42% less likely to achieve a live birth (*β* = − 0.4172361, *p* = 0.052).

**Conclusions:**

Individuals accessing preimplantation genetic testing for monogenic conditions have higher clinical pregnancy and live birth rates than couples accessing in vitro fertilization for other indications such as subfertility. These findings confirm that preimplantation genetic testing is an effective reproductive option for Australian carrier individuals.

**Supplementary Information:**

The online version contains supplementary material available at 10.1007/s10815-025-03416-6.

## Introduction

Monogenic conditions are caused by a variation in a single gene [1]. Collectively, these conditions occur in ~ 1 in 250 births and contribute significantly to global morbidity and mortality [2–5].

When an individual is identified with an increased chance of having a child with a monogenic condition, they may consider several reproductive options. These include using the available information to prepare for the birth of a child with a monogenic condition, adoption, using donor gametes, prenatal diagnostic testing, and/or preimplantation genetic testing (PGT-M) [6].

PGT-M is a technique used to characterise the genetic status of cells biopsied from embryos created in vitro [7]. Embryos categorised as having a low chance of the condition of interest can then be selectively transferred to the uterus [8]. This approach enables the conception of a biologically related child while minimising the chance of the condition of interest in the pregnancy.

Providing clear information on PGT-M clinical outcomes is a key component of pre-test counselling, facilitating informed decision-making and enabling prospective patients to set realistic expectations. While many fertility centres internationally have reported clinic-specific outcomes following PGT-M, there are no existing studies reporting outcomes from clinics in the Oceanic region, including Australian-based clinics [9–15].

It is difficult to use international evidence to inform pre-test counselling of individuals accessing PGT-M in an Australian context due to the many local factors that impact clinical outcomes [16]. This includes the availability of highly trained staff and sophisticated technology, as well as patient-specific variables including age, weight, consumption of alcohol and tobacco, and ability to afford treatment [17, 18]. It is also important to continue evaluation of PGT-M to provide patients with current outcome figures to accurately inform decision-making.

We aimed to address this evidence gap by performing a retrospective study of outcomes of PGT-M cycles conducted between 2015 and 2022 at a large Australian IVF provider with clinics in two states. The primary objective was to evaluate live birth rates per embryo transfer in individuals where the primary indication for assisted reproduction was PGT-M. We also compared the live birth rates after IVF for PGT-M with that of our centre’s general IVF population.

## Materials and methods

This retrospective study collected data on PGT-M cycles performed between January 2015 and December 2022 at Monash IVF, a large private fertility centre in Australia. Only cycles that resulted in at least one embryo biopsied for PGT for a monogenic indication were included in the analysis. Patients from two Australian states (Victoria and Queensland) were included.

### Referral pathway, and clinical and laboratory protocols

The following methodology describes standard clinical procedures associated with PGT-M at the centre over the study period.

### Patient population

The centre’s genetic counselling team initially assessed all referrals. All patients received pre-test counselling from their fertility specialist, clinical geneticist, and/or genetic counsellor. Written consent was obtained from all couples before commencing the cycling process. After confirming the genetic status, DNA samples were collected from each couple, as well as from one or more relatives, to facilitate feasibility studies.

### Stimulation, retrieval, biopsy, and fertilisation procedure

The ovarian stimulation, oocyte retrieval, and fertilisation procedures followed published protocols [19, 20]. In short, 95% of patients underwent a GnRH antagonist cycle (Orgalutran®; Ganirelix; Merck Sharp & Dohme, Macquarie Park, Australia) with recombinant FSH (Gonal-F®; Merck Serono, Frenchs Forest, Australia; Puregon; Merck Sharp & Dohme, South Granville, Australia). Oocyte retrieval (oocyte pick up (OPU)) was scheduled 35 or 36 h after the human chorionic gonadotrophin (hCG) trigger. All oocytes were fertilised using intracytoplasmic sperm injection (ICSI) 40 h post trigger.

### Embryo culture, testing, transfer, and freezing

After ICSI, the clinical embryologists cultured the embryos and assessed fertilisation at 16-18h [20]. Embryos were assessed, developmentally classified and quality graded as previously described [20, 21]. Embryos were considered suitable for biopsy between days 5 and 7 if they contained a clearly defined inner cell mass and a suitable number of healthy trophectoderm cells (≥ 30). Approximately five trophectoderm cells for PGT-M were biopsied using a combination of laser and mechanical biopsy techniques. Whole genome amplification was performed on biopsy samples using the RepliG Single Cell Kit (Qiagen, the Netherlands). Karyomapping protocol was performed at the fertility centre’s genetics laboratory as previously described [22]. The use of karyomapping enables haplotype phasing and simultaneous 24-chromosome screening (known as preimplantation genetic testing for aneuploidy, or PGT-A).

### Embryo transfer

On day 5, up to two frozen-thawed embryos were transferred, either in a natural cycle or a hormone replacement cycle [19]. The clinic follows a single embryo transfer policy; however, double embryo transfers were occasionally performed at patient request, in recognition of patient autonomy. Human chorionic gonadotrophin (hCG) testing was undertaken ~ 14 days post embryo transfer to confirm biochemical pregnancy status. A viability ultrasound is performed between 7 and 9 weeks post-embryo transfer to confirm clinical pregnancy status. Following confirmation of the pregnancy, patients are referred by their fertility specialist for obstetric care.

### Birth outcomes

Treating obstetricians provided birth outcomes, including date of delivery, gestational age, weight, and any birth complications, or infant health concerns, to the fertility centre in compliance with the licensing requirements of the Reproductive Technology Accreditation Committee (RTAC). The RTAC Code of Practice mandates reporting of all pregnancy outcomes to the Australian and New Zealand Assisted Reproduction Database (ANZARD) for clinical quality monitoring. Postnatal testing is not routinely performed for all monogenic indications. This is particularly true of conditions with adult age of onset. Postnatal testing outcomes were subsequently unavailable, and a misdiagnosis rate was therefore unable to be ascertained.

### Definition of data terms

Please see Table 1 for a definition of data terms used within this audit.

**Table 1:** Definition of data terms

Term	Definition
Cycle	Any ovarian stimulation protocol that resulted in at least one embryo available for biopsy
Embryo transferred	A single embryo that has been transferred within an embryo transfer cycle
Embryo transfer cycle	A frozen embryo transfer, whereby up to 2 embryos may be transferred at a time
Clinical pregnancy	As per the Australian and New Zealand Assisted Reproductive Database (ANZARD) as a pregnancy that fulfils one of the following criteria: • “Known to be ongoing at 20 weeks; • Evidence by ultrasound detection of an intrauterine sac (with or without a fetal heart); • Examination of products of conception reveal chorionic villi; • Or an ectopic pregnancy that has been diagnosed by laparoscope or by ultrasound”
Live birth	As per the World Health Organisation (WHO)
Early pregnancy loss	Any clinical pregnancy loss before 20 weeks gestation. This term includes miscarriage, ectopic and molar pregnancies
Still birth	The death of a fetus before or during birth after 28 weeks gestation
Neonatal death	The death of a baby within the first 28 days of life
Subfertility indication	Any subfertility covariate listed by the patient’s referring clinician, at the clinician’s discretion. The list of indications is available in supplementary Table 2
Suitable for transfer	An embryo that was either low risk for the condition of interest, or a recessive carrier of the condition of interest, and euploid
Not suitable for transfer	An embryo that was either affected by the condition of interest and/or aneuploid
Suitable for transfer following additional consultation or testing	Embryos, such as those with an inconclusive result, biopsy performed but testing not yet performed, low-moderate chromosomal mosaicism, or a female carrier of an X-linked dominant condition. These embryos may be considered suitable for transfer following consultation with a specialist, or if additional testing is performed
Inconclusive	Embryo results including those that are inconclusive due to poor quality data, recombination events in the gene region of interest, and aneuploidy involving the gene region of interest
Biopsy taken but testing not performed	Embryos that were biopsied and have had DNA amplified using RepliG, however, the karyomapping protocol has not yet been performed. Since 2021, in Australia, government rebates have been available for the testing of ≤ three embryos. Individuals with ≥ three embryos suitable for biopsy may subsequently elect to biopsy additional embryos, without performing analysis, due to financial considerations
Result pending	Analysis that has begun on embryo biopsies, however results were not available at time of reporting

### Data collection

We collected data from the fertility centre’s Regulatory Information Management Software, which stores medical records held as part of clinical care. These records include the results of PGT, which are routinely entered into the software by the laboratory’s genetic scientists. This dataset was searched for all instances of PGT-M/A during the study time frame, and relevant data was extracted. We stored and analysed data using Microsoft Excel and STATA v.18.

We extracted data on PGT-M/A outcomes including the results of monogenic and aneuploidy screening, the number of stimulated cycles resulting in embryo biopsy, the number of embryo transfers, and the number of clinical pregnancies and live births.

### Data analysis

We calculated clinical pregnancy and live birth rates using three denominators: total cycles, total transferred embryos, and total embryo transfer cycles. We also stratified data according to the monogenic inheritance pattern, and rates were calculated within these groups.

We also analysed the relationship between binary clinical outcomes (clinical pregnancy and live birth) and fertility covariates (body mass index, maternal age, FSH Dose, and the presence of a subfertility indication) within the PGT-M/A cohort. To account for the potential correlation within patients who have undergone multiple PGT-M cycles, Generalised Estimating Equations (GEE) were used to analyse these relationships. We selected a binomial distribution with a logit link function. We assumed the exchangeable correlation structure to accommodate possible intra-patient correlation. We estimated the GEE model using Stata V.18 [23].

### Comparison groups

Two comparison groups were selected to evaluate PGT-M/A outcomes. The first group included PGT-A only tested embryo transfers between 2015 and 2022. The second group consisted of all frozen embryo transfers undertaken at the fertility centre during the same period. This 8-year time frame aligns with the study period of the PGT-M/A data.

The PGT-A only comparison group was selected as all PGT-M tested embryos undergo concurrent aneuploidy screening at our centre, allowing for a comparison that partly controls for aneuploidy. Clinical outcome data for the PGT-A tested embryo transfers, including information on maternal age at the time of treatment, were collected from the fertility centre’s Regulatory Information Management Software. To enable age matching with the PGT-M/A cohort, outcomes were stratified into “under 35” and “35 and over” groups. This stratification was selected as 35 is widely recognised as the age threshold of advanced maternal age. Age stratification was performed to control for the potential confounding effects of maternal age, allowing an assessment of whether other factors may contribute to observed differences between the groups.

The frozen embryo transfer group, which includes both PGT-tested and untested embryos, was selected as the general IVF comparison group. Within clinical practice, general IVF outcome data are often presented to prospective PGT-M/A patients in lieu of PGT-M/A-specific figures. A general IVF comparison group was therefore included to enable the comparison of PGT-M/A outcomes with those typically presented to patients. Clinical outcome data following the transfer of frozen embryos were extracted from annual reports from the Victorian Assisted Reproductive Treatment Authority (VARTA) [24]. This included information on the age distribution of individuals undergoing treatment.

To test for differences between the clinical outcomes of the comparison groups and the PGT-M/A cohort, an N-1 chi-square test was performed using the MedCalc Comparisons of Proportions Calculator.

The group undergoing aneuploidy screening alone during the study period (PGT-A only) had aneuploidy screening using low coverage whole genome sequencing (Illumina Veriseq Solution) as per manufacturer’s instructions. In contrast, the group having aneuploidy screening performed concurrent with PGT-M (in the PGT-M/A group) had aneuploidy screening performed by manual analysis of haplotypes and logR/BAF visualisation using the karyomapping protocol (Illumina) according to an in-house standard operating procedure. Data on the origins of chromosomal aneuploidy were obtained using the karyomapping protocol; however, this was limited to identifying meiotic or high-level mitotic origins, with the distinction between them not reported. As a result, the karyomapping protocol exhibits lower sensitivity for detecting low-level mosaicism. Both methods are capable of detecting segmental aneuploidy to a resolution of 10Mb. While these methods differ in their aneuploidy calling criteria, both are commonly used clinically in the detection of whole chromosome aneuploidy.

## Results

During the 8-year study period, 572 PGT-M/A cycles were performed for 299 patients, with a mean of 1.9 cycles per patient. The mean maternal age at the time of oocyte collection was 33.7, and the mean maternal BMI was 25.0. A subfertility indication was recorded in 268 cycles (46.8%). A list of subfertility indications recorded is presented in Supplementary Table 1. Patients received an FSH dosage of 300 IU or higher in 166 cycles (29.0%). The most common indications for testing were cystic fibrosis, fragile X syndrome, and myotonic dystrophy type 1.

### Outcomes

**Fig. 1:**
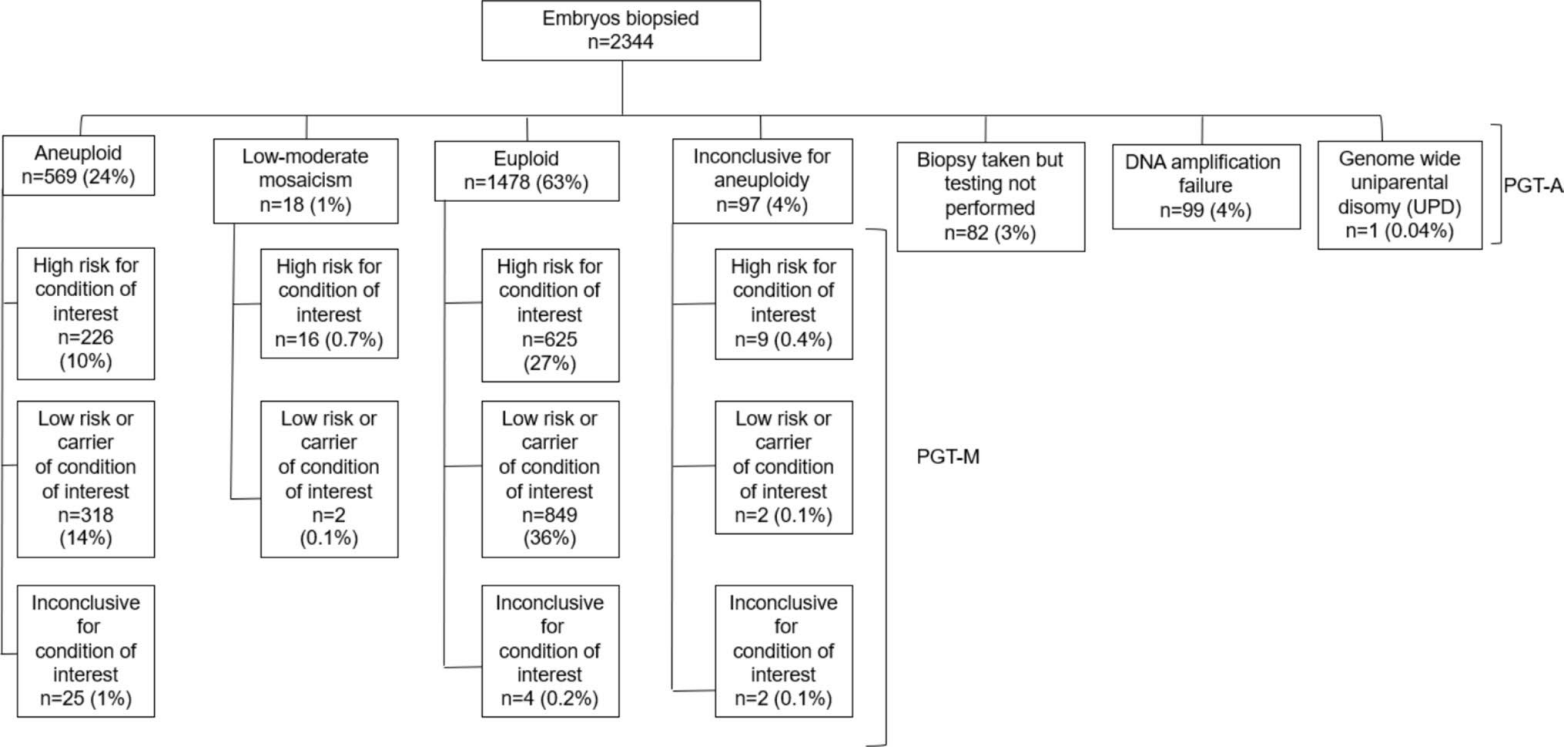
Testing outcomes following embryo biopsy

These 572 cycles resulted in 2344 embryo biopsies. Of those biopsied, 849 (36.2%) were suitable for transfer, 1219 (52.0%) were not suitable for transfer, and 276 (11.8%) were suitable for transfer following additional consultation or testing. Figure 1 presents the testing outcomes. Supplementary Table 2 provides detailed outcome data. Out of the 572 cycles, 392 cycles (68.5%) had at least one suitable embryo for transfer, and 355 cycles (62.1%) resulted in at least one embryo transfer.

#### Descriptive caption

Flowchart depicting the outcomes of 2344 embryos biopsied for preimplantation genetic testing. The embryos were categorized based on aneuploidy status: aneuploid (569, 24%); low-moderate mosaicism (18, 1%); euploid (1478, 63%); inconclusive for aneuploidy (97, 4%); biopsy taken but testing not performed (82, 3%); DNA amplification failure (99, 4%); and genome-wide uniparental disomy (UPD) (1, 0.4%). Each category is further divided based on the risk for the condition of interest and the conclusive or inconclusive results for the condition, illustrating the distribution and diagnostic outcomes for preimplantation genetic testing for monogenic conditions (PGT-M) and aneuploidy (PGT-A).

#### Clinical pregnancy and live birth rates

**Fig. 2:**
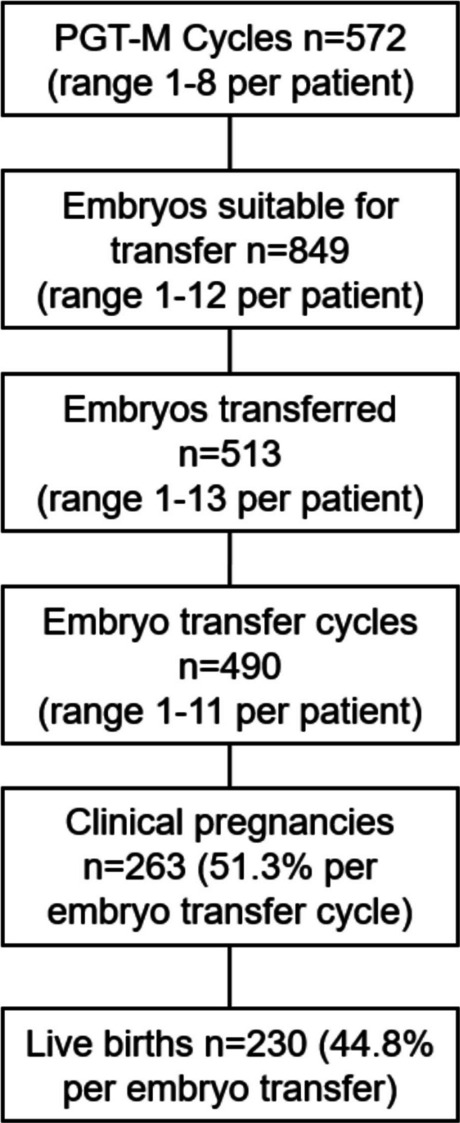
Clinical pregnancies and live births following PGT-M/A

Clinical outcomes are presented in Fig. 2. Clinical pregnancies and live birth rates are presented in Table 2.

**Table 2:** Clinical pregnancy and live birth rates following PGT-M

	Clinical pregnancy rate		Live birth rate	
	n/N	% [95% CI]	n/N	% [95% CI]
**Per stimulated cycle**	263/572	46.0 [41.9–50.1]	230/572	40.2 [36.3–44.3]
**Per embryo transferred**	263/513	51.3 [47.0–55.6]	230/513	44.8 [40.6–49.2]
**Per embryo transfer cycle**	263/490	53.7 [49.3–58.0]	230/490	46.9 [42.6–51.4]

#### Descriptive caption

Flowchart detailing the outcomes of 572 PGT-M/A cycles. Out of 849 embryos deemed suitable for transfer, 513 were transferred across 490 embryo transfer cycles. This resulted in 263 clinical pregnancies and 230 live births, illustrating the progression from PGT-M/A cycles to live birth outcomes.

### Outcomes per monogenic indication

Of the 572 cycles, 159 (27.8%) were for autosomal recessive indications, 293 (51.2%) were for autosomal dominant indications, 64 (11.2%) were for X-linked recessive indications, and 56 (9.8%) were for X-linked dominant indications. During the study period, PGT-M was accessed for 131 monogenic conditions. A complete list of conditions tested for is not reported due to potential privacy converns.

Of the 2344 embryos biopsied for PGT-M/A, 673 were tested for autosomal recessive indications, 1259 were tested for autosomal dominant indications, 263 were tested for X-linked recessive indications, and 149 were tested for X-linked dominant indications. A detailed summary of the clinical recommendation following biopsy for each indication group is presented in Table 3. Demographic information is presented in Supplementary Table 3, raw clinical outcome figures are presented in Supplementary Table 4 and embryo testing outcomes are presented in Supplementary Tables 5 and 6.

**Table 3:** Clinical recommendations for PGT-M tested embryos by monogenic inheritance pattern

Inheritance pattern	Suitable for transfer	Not suitable for transfer	Suitable for transfer following additional consultation or testing	Total
Autosomal recessive	284 (42.2%)	297 (44.1%)	92 (13.7%)	673
Autosomal dominant	378 (30.0%)	759 (60.3%)	122 (9.7%)	1259
X-linked recessive	124 (47.1%)	93 (35.4%)	46 (17.5%)	263
X-linked dominant	63 (42.3%)	70 (47.0%)	16 (10.7%)	149
Total	849	1219	276	2344

Clinical pregnancy and live birth rates per monogenic inheritance pattern are presented in Table 4. Cycles and embryo transfers completed for autosomal recessive indications achieved the highest comparative clinical pregnancy and live birth rates. Cycle-based rates for X-linked dominant conditions were comparatively low.

**Table 4:** Clinical pregnancy and live birth rates following PGT-M by monogenic inheritance pattern

Inheritance type	Outcome denominator	Clinical pregnancy rate		Live birth rate	
		n/N	% [95% CI]	n/N	% [95% CI]
**Autosomal recessive**	**Per stimulated cycle**	82/159	51.6 [43.9–59.2]	74/159	46.5 [39.0–54.3]
	**Per embryos transferred**	82/152	54.0 [46.0–61.7]	74/152	48.7 [40.9–56.6]
	**Per embryo transfer cycle**	82/145	56.6 [48.4–64.4]	74/145	51.0 [43.0–59.0]
**Autosomal dominant**	**Per stimulated cycle**	130/293	44.4 [38.8–50.1]	114/293	38.9 [33.5–44.6]
	**Per embryos transferred**	130/254	51.2 [45.1–57.3]	114/254	44.9 [38.9–51.0]
	**Per embryo transfer cycle**	130/245	53.1 [46.8–59.2]	114/245	46.5 [40.4–52.8]
**X-linked recessive**	**Per stimulated cycle**	31/64	48.4 [36.6–60.4]	26/64	40.6 [29.5–52.9]
	**Per embryos transferred**	31/67	46.3 [34.9–58.1]	26/67	38.8 [28.1–50.8]
	**Per embryo transfer cycle**	31/62	50.0 [37.9–62.1]	26/62	41.9 [30.5–54.3]
**X-linked dominant**	**Per stimulated cycle**	20/56	35.7 [24.5–48.8]	17/56	30.4 [19.9–43.3]
	**Per embryos transferred**	20/40	50.0 [35.2–64.8]	17/40	42.5 [28.5–57.8]
	**Per embryo transfer cycle**	20/38	52.6 [37.3–67.5]	17/38	44.7 [30.2–60.3]

### Generalized estimating equations (GEE) analysis of clinical outcomes within the PGT-M/A cohort

Compared with PGT-M/A patients without a subfertility factor, PGT-M/A patients with a subfertility factor were 48% less likely to achieve a clinical pregnancy per embryo transfer (*β* coefficient = − 0.48, *p* = 0.026) and 42% less likely to achieve a live birth (*β* coefficient = − 0.42, *p* = 0.052).

There was a trend toward lower likelihoods of clinical pregnancies and live births per PGT-M/A cycle among individuals of advanced maternal age (*β* coefficient = − 0.38, *p* = 0.067 and *β* coefficient = − 0.40, *p* = 0.06). Lower likelihood of clinical pregnancy and live birth rates per PGT-M/A cycle were also noted for individuals of low or high BMI, or those with an initial FSH dosage of > 300 IU; however, this was nonsignificant (*p* > 0.1 for all outcomes). The detailed outcomes of GEE analysis are provided in Supplementary Tables 7 and 8.

### Comparison of PGT-M/A and PGT-A outcomes–age-stratified analysis

PGT-A tested embryos: During the study period, 5339 PGT-A tested embryos were transferred, resulting in 2617 clinical pregnancies and 2212 live births. This gave clinical pregnancy and live birth rates per embryo transfer of 49.0% and 41.4%, respectively. These rates were not different from the corresponding rates of 51.3% (*p* = 0.3196) and 44.8% (*p* = 0.1358) in our PGT-M cohort. However, when stratified by maternal age, a difference was observed in individuals aged 35 and over, with the PGT-M/A group showing higher clinical pregnancy (57.7% vs 48.6%, *p* = 0.04) and live birth rates (51.1% vs 40.8% *p* = 0.02) than the PGT-A only group. Clinical outcomes of both groups are provided in Table 5.

**Table 5:** Clinical outcomes of PGT-M/A and PGT-A stratified by age

	Under 35 years					35 years and over				
	PGT-A		PGT-M/A		*P* value*	PGT-A		PGT-M/A		*P* value*
Mean maternal age	32.2 years		30.7 years			38.2 years		37.2 years		
	n/N	%	n/N	%		n/N	%	n/N	%	
Clinical pregnancy rate per embryo transfer	905/1816	49.8	184/376	48.9	0.7508	1712/3523	48.6	79/137	57.7	0.0366
Live birth per embryo transfer	774/1816	42.6	160/376	42.6	1	1438/3523	40.8	70/137	51.1	0.0163

### Comparison of PGT-M/A and frozen embryo transfer outcomes

**Fig. 3:**
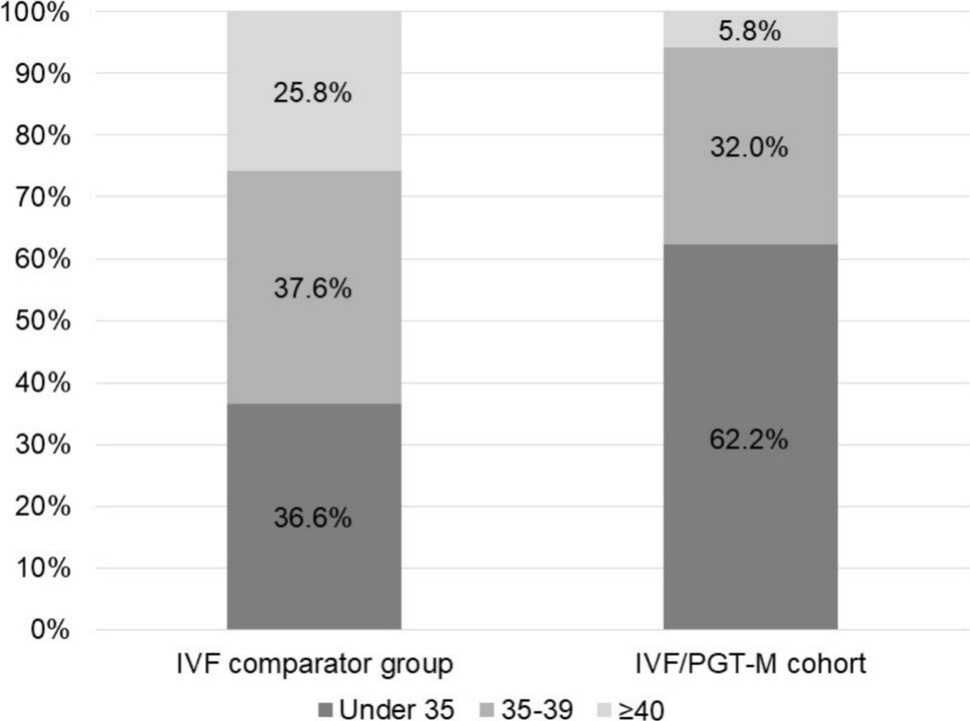
Maternal age distribution of IVF and ICSI/PGT-A cohorts and PGT-M/A

Between 2015 and 2022, VARTA reported 18229 frozen embryo transfers at the fertility centre’s Victorian clinics. These resulted in 7107 clinical pregnancies and 5755 live births. This gave clinical pregnancy and live birth rates per embryo transfer of 39.0% and 31.6%, respectively. These rates are significantly lower than the corresponding rates in our PGT-M/A cohort (*p* < 0.0001). The maternal age distribution of this PGT-M/A cohort and the comparator cohort is shown in (Fig. 3).

Descriptive caption: Stacked bar chart comparing age distributions between two groups: the IVF comparator group and the IVF/PGT-M/A cohort. In the IVF comparator group, the age distribution is 36.6% under 35, 37.6% aged 35 − 39, and 25.8% aged 40 or above. In the IVF/PGT-M/A cohort, the age distribution is 62.2% under 35, 32.0% aged 35 − 39, and 5.8% aged 40 or above.

## Discussion

### Overall findings

In this comprehensive analysis of PGT-M/A outcomes from one of Australia’s largest fertility providers, we report higher clinical pregnancy and live birth rates than those previously reported in the international literature [25]. The clinical pregnancy and live birth rates per embryo transfer of 51.3% and 44.8% are also higher than outcomes for our general IVF population. These improved rates may be attributed to a population with lower rates of subfertility factors accessing PGT-M/A, including, advanced maternal age. Within the fertility centre’s PGT-M/A cohort, 37.8% of patients were ≥ 35 years of age, compared to 63.4% in the local general IVF population. Increased maternal age is well-reported to have a negative impact on IVF clinical outcomes, including within PGT-M/A cohorts [26].

Aneuploidy is recognised as a major contributor to declining clinical outcomes with advancing maternal age. However, when partly controlling for aneuploidy by comparing the PGT-M/A cohort to an age-matched PGT-A cohort, improved outcomes were still observed in individuals over 35. This suggests that improved rates in the PGT-M/A population may not only be attributed to a younger patient population, but to additional factors including other subfertility factors. Stimulation regiment may also have contributed to different endpoint measure outcomes. Only 29.0% of individuals within our PGT-M/A cohort received a high FSH dosage, suggesting the majority of patients were not expected to be low responders. This differs from the general IVF population, where around half of patients are typically expected to be on the maximum FSH dose [27].

Additionally, less than half of our PGT-M/A cohort had a subfertility indication recorded, suggesting that ART utilization was most commonly motivated by genetic status alone. Conversely, within a general ART population, it is presumed individuals are largely accessing treatment due to a subfertility indication. This would be expected to impact clinical outcomes, as seen in our GEE analysis, which reported a decreased likelihood of clinical pregnancy and live birth in patients with a recorded subfertility factor, and likely explains the differences noted between our cohort and the local general IVF and PGT-A populations. These differences demonstrate the limitations in providing general IVF outcome figures to PGT-M/A cohorts.

We also found that clinical pregnancy and live birth rates were higher in cycles undertaken for recessive conditions than for dominant conditions. This outcome was anticipated, as dominant conditions should statistically result in a higher number of high-risk embryos, and subsequently a greater proportion of embryos unsuitable for transfer (Wiacker & Steinhard, 2010).

X-linked dominant conditions had the lowest rate of success among all inheritance patterns. The most common indication for testing within this group was Fragile X syndrome. Fragile X carriers have diminished ovarian reserve, and this is a possible explanation for the lower IVF success rates [28].

### Implications for clinical practice

This is the first detailed analysis of PGT-M clinical outcomes in an Australian population. It provides valuable data to inform evidence-based counselling of patients considering their reproductive options and facilitate shared decision-making. It confirms widespread consumer and clinician expectations of better clinical outcomes for the PGT-M population compared with the general ART population.

Our results are timely as changes in carrier screening funding in Australia are expected to increase the detection of carrier couples in the near future. Carrier screening for cystic fibrosis, fragile X syndrome, and spinal muscular atrophy became publicly funded by Australia’s healthcare system in 2023 [29]. Publicly-funded carrier screening is accompanied by government funding for IVF for PGT-M, either through rebates for private clinic services or through direct provision in public hospital fertility units. The removal of financial barriers to screening and reproductive options is anticipated to increase demand for PGT-M in Australia [30]. This changing landscape highlights the importance of having relevant and up-to-date data to inform accurate counselling and informed decision making of prospective patients [31, 32].

Our findings also delineate the variation in clinical outcomes according to inheritance pattern. This emphasises the importance of providing individualised genetic counselling according to the relevant inheritance pattern. Counselling should also incorporate fertility covariates, including maternal age and subfertility indications. The results underscore the importance of completing a standard fertility evaluation for prospective PGT-M couples to allow informed decision-making about their reproductive options.

### Limitations

Our findings were limited by the unavailability of information about cycles that did not proceed to the embryo biopsy stage. Information on the number of cancelled cycles, or cycles that obtained no embryos suitable for biopsy are not reported in the current study. It is important to interpret rates per cycle within this context.

The absence of an AMH level within our dataset is a limitation of our multivariate analysis. AMH is well-reported as a predictor of reproductive potential [33]. While our dataset includes FSH start dose per cycle, which can indicate AMH levels, FSH start dosage is determined based on clinical judgement, which can incorporate additional factors such as age and previous response to stimulation [34].

Subfertility indications were recorded by the treating clinician and were not subjected to a data quality assessment. There subsequently may be variability surrounding the classification and recording of subfertility indications.

While this study presents valuable data on PGT-M within an Australian setting, clinic-specific differences may limit the generalisability of these findings. However, our IVF and PGT-M protocols are consistent with international standards and thus may still be applicable to other settings.

The variation in aneuploidy calling criteria across the different platforms utilised by our PGT-A and our PGT-M/A groups may affect the classification of embryos as euploid, aneuploid, or mosaic, potentially influencing clinical outcomes. Nevertheless, this comparison remains relevant as it reflects real-world clinical practices, where diverse aneuploidy screening methods are employed across different indication groups. It also offers the most appropriate match currently available in terms of biopsy protocol and the inclusion of aneuploidy screening.

### Future research

It would be valuable to examine the relationship between clinical outcomes and fertility variables, such as AMH, in a larger sample in order to provide more accurate prognostic information for couples with concurrent subfertility factors.

Future research could also examine strategies to incorporate these findings into clinical practice, including the development of decision aids, or clinical prediction tools to assist informed decision-making surrounding the access of PGT-M.

We did not explore any aspect of the consumer experience in this study. We have now commenced qualitative research to provide additional insights to improve the clinical care of this population.

## Conclusions

Clinical pregnancy and live birth rates following PGT-M are higher than the general IVF and ICSI population, despite the need to exclude affected embryos. These positive clinical outcomes reflect the more favourable fertility profile of the couples accessing PGT-M. These data will help inform the genetic counselling of carrier couples who are considering PGT-M among their reproductive options.

## Supplementary Information

Below is the link to the electronic supplementary material.

Supplementary file1 List of subfertility indications categorized by female and male factors. Female subfertility indications include diminished ovarian reserve (diminished ovarian reserve, Fragile X carrier, Fragile X carrier with low AMH, low AMH), increased ovarian reserve (polycystic ovaries, polycystic ovary syndrome), ovulation disorders, factors impacting embryo implantation (endometriosis, unexplained endometriosis, uterine fibroids), unexplained subfertility (idiopathic), and other factors (chromosome mosaic, previous ectopic pregnancy). Male subfertility indications include factors impacting sperm production (azoospermia), sperm shape (oligoasthenoteratozoospermia, teratozoospermia), obstructive causes (congenital absence of the vas deferens, cystic fibrosis carrier), unexplained subfertility (idiopathic), and other factors (testicular cancer). (PDF 47 KB)

Supplementary file2 Detailed embryo testing outcomes for PGT-A and PGT-M cycles. The table presents the outcomes of 2344 embryos tested, categorized by the presence of aneuploidy, inconclusive results, mosaicism, euploid status, biopsy taken but testing not performed, result pending, and DNA amplification failure. Outcomes are further detailed by the risk for the condition of interest: high risk (876 embryos), low risk (843 embryos), carrier status (330 embryos), inconclusive due to aneuploidy in the region of interest (22 embryos), and other inconclusive results (92 embryos). Additional categories include biopsy taken but testing not performed (77 embryos), result pending (5 embryos), and DNA amplification failure (99 embryos). (PDF 47.5 KB)

Supplementary file3 Demographic information categorized by inheritance pattern. The table includes maternal age (mean and median per cycle), the top three conditions per cycle, subfertility flagged, subfertility impacting ovarian reserve, subfertility impacting embryo implantation per cycle, and high FSH start dose. For autosomal recessive conditions, the mean maternal age is 34.30 and the median is 34.34, with CF, beta thalassemia, and SMA being the top conditions. For autosomal dominant conditions, the mean maternal age is 33.25 and the median is 32.84, with myotonic dystrophy, Huntington’s disease, and familial chromosomal micro deletion being the top conditions. For X-linked recessive conditions, the mean maternal age is 33.40 and the median is 32.815, with DMD, haemophilia, and Wiskott-Aldrich syndrome being the top conditions. For X-linked dominant conditions, the mean maternal age is 34.38 and the median is 34.515, with Fragile X, RP 3, and CMT XLD type 1 being the top conditions. Subfertility impacting ovarian reserve, embryo implantation, and high FSH start dose are also detailed for each inheritance pattern. (PDF 86 KB)

Supplementary file4 Clinical outcomes categorized by monogenic inheritance pattern. The table includes the number of cycles, embryos biopsied, embryos transferred, embryo transfer cycles, clinical pregnancies, and births (including live births and ongoing pregnancies) for each inheritance pattern: autosomal recessive, autosomal dominant, X-linked recessive, and X-linked dominant. For autosomal recessive conditions, there were 159 cycles, 673 embryos biopsied, 152 embryos transferred, 145 embryo transfer cycles, 82 clinical pregnancies, and 74 births. For autosomal dominant conditions, there were 293 cycles, 1259 embryos biopsied, 254 embryos transferred, 245 embryo transfer cycles, 130 clinical pregnancies, and 114 births. For X-linked recessive conditions, there were 64 cycles, 263 embryos biopsied, 67 embryos transferred, 62 embryo transfer cycles, 31 clinical pregnancies, and 26 births. For X-linked dominant conditions, there were 56 cycles, 149 embryos biopsied, 40 embryos transferred, 38 embryo transfer cycles, 20 clinical pregnancies, and 17 births. (PDF 36 KB)

Supplementary file5 Aneuploidy screening outcomes categorized by monogenic inheritance pattern. The table presents the outcomes of aneuploidy testing for embryos classified as autosomal recessive, autosomal dominant, X-linked recessive, and X-linked dominant. The outcomes include the number and percentage of embryos found to be aneuploid, with low-moderate mosaicism, euploid, inconclusive, biopsy taken but testing not performed, DNA amplification failure, and result pending. For autosomal recessive conditions, 170 embryos (25.3%) were aneuploid, 6 (0.9%) had low-moderate mosaicism, 405 (60.2%) were euploid, 27 (4.0%) were inconclusive, 29 (4.3%) had biopsy taken but testing not performed, and 36 (5.3%) experienced DNA amplification failure. For autosomal dominant conditions, 313 embryos (24.9%) were aneuploid, 8 (0.6%) had low-moderate mosaicism, 810 (64.3%) were euploid, 50 (4.0%) were inconclusive, 36 (3.0%) had biopsy taken but testing not performed, and 42 (3.3%) experienced DNA amplification failure. For X-linked recessive conditions, 52 embryos (19.8%) were aneuploid, 4 (1.5%) had low-moderate mosaicism, 164 (62.4%) were euploid, 16 (4.0%) were inconclusive, 8 (2.9%) had biopsy taken but testing not performed, and 15 (3.3%) experienced DNA amplification failure. For X-linked dominant conditions, 52 embryos (20.0%) were aneuploid, 4 (1.5%) had low-moderate mosaicism, 164 (63.1%) were euploid, 16 (6.2%) were inconclusive, 8 (3.1%) had biopsy taken but testing not performed, 15 (5.8%) experienced DNA amplification failure, and 1 (0.4%) had a result pending. (PDF 38.3 KB)

Supplementary file6 Monogenic screening outcomes categorized by monogenic inheritance pattern. This table provides the results of monogenic testing for embryos classified under autosomal recessive, autosomal dominant, X-linked recessive, and X-linked dominant inheritance patterns. The outcomes include the number and percentage of embryos identified as high risk for the condition of interest, low risk for the condition of interest, carrier (or affected female embryo for X-linked dominant), inconclusive due to aneuploidy in the region of interest, inconclusive, biopsy taken but testing not performed, result pending, and DNA amplification failure. For autosomal recessive conditions, 167 embryos (24.8%) were high risk, 136 (20.2%) were low risk, 270 (40.1%) were carriers, 8 (1.2%) were inconclusive due to aneuploidy in the region of interest, 27 (4.0%) were inconclusive, 29 (4.3%) had biopsy taken but testing not performed, and 36 (5.3%) experienced DNA amplification failure. For autosomal dominant conditions, 604 embryos (48.0%) were high risk, 525 (41.7%) were low risk, 9 (0.7%) were inconclusive due to aneuploidy in the region of interest, 43 (3.4%) were inconclusive, 36 (2.9%) had biopsy taken but testing not performed, and 42 (3.3%) experienced DNA amplification failure. For X-linked recessive conditions, 54 embryos (20.5%) were high risk, 120 (45.6%) were low risk, 43 (16.3%) were carriers, 2 (0.8%) were inconclusive due to aneuploidy in the region of interest, 17 (6.5%) were inconclusive, 8 (3.0%) had biopsy taken but testing not performed, 4 (1.5%) had a result pending, and 15 (5.7%) experienced DNA amplification failure. For X-linked dominant conditions, 51 embryos (34.2%) were high risk, 62 (41.6%) were low risk, 17 (11.4%) were carriers or affected female embryos, 3 (2.0%) were inconclusive due to aneuploidy in the region of interest, 5 (3.4%) were inconclusive, 4 (2.7%) had biopsy taken but testing not performed, 1 (0.7%) had a result pending, and 6 (4.0%) experienced DNA amplification failure. (PDF 38.6 KB)

Supplementary file7 This table summarizes the GEE analysis outcomes for clinical pregnancy and live birth rates per cycle, based on 449 observations from 229 groups. The number of observations per group ranges from a minimum of 1 to a maximum of 8, with an average of 2.0 observations per group. For clinical pregnancy, the Wald chi-squared statistic is 7.60 with a p-value of 0.1075, indicating no statistically significant predictors. The coefficients, standard errors, and p-values for the predictors are as follows: advanced maternal age (coefficient = -0.3888, *p* = 0.067), BMI high or low (coefficient = -0.1761, *p* = 0.393), FSH start dose 300 or above (coefficient = -0.1507, *p* = 0.486), and subfertility indication flagged (coefficient = 0.3133, *p* = 0.127). For live birth, the Wald chi-squared statistic is 7.56 with a p-value of 0.1091, also indicating no statistically significant predictors. The predictors' coefficients, standard errors, and p-values are as follows: advanced maternal age (coefficient = -0.4047, *p* = 0.061), BMI high or low (coefficient = -0.1147, *p* = 0.582), FSH start dose 300 or above (coefficient = -0.2422, *p* = 0.274), and subfertility indication flagged (coefficient = 0.2542, *p* = 0.221). (PDF 40 KB)

Supplementary file8 This table summarizes the GEE analysis outcomes for clinical pregnancy and live birth rates per embryo transferred, based on 421 observations from 193 groups. The number of observations per group ranges from a minimum of 1 to a maximum of 13, with an average of 2.2 observations per group. For clinical pregnancy, the Wald chi-squared statistic is 7.12 with a p-value of 0.1296, indicating no statistically significant predictors. The coefficients, standard errors, and p-values for the predictors are as follows: advanced maternal age (coefficient = 0.3691, *p* = 0.117), BMI high or low (coefficient = 0.0471, *p* = 0.824), FSH start dose 300 or above (coefficient = -0.0850, *p* = 0.715), and subfertility indication flagged (coefficient = -0.4797, *p* = 0.026). For live birth, the Wald chi-squared statistic is 5.76 with a p-value of 0.2177, also indicating no statistically significant predictors. The predictors' coefficients, standard errors, and p-values are as follows: advanced maternal age (coefficient = 0.3407, *p* = 0.145), BMI high or low (coefficient = 0.0726, *p* = 0.733), FSH start dose 300 or above (coefficient = -0.0680, *p* = 0.771), and subfertility indication flagged (coefficient = -0.4172, *p* = 0.052). (PDF 39 KB)

Supplementary file9 (PDF 95 KB)

## Data Availability

The data underlying this article are not publicly available due to the conditions of human research ethics approval. Non-identifiable participant data from this study are available to researchers affiliated with a recognized academic institution, upon reasonable request to the corresponding author. The study investigators may contribute aggregate and non-identifiable individual patient data to researchers whose proposed use of the data has been ethically reviewed and approved by an independent committee, and following the signing of an appropriate research collaboration agreement.
