# Supplementary material for: IVF success rates in individuals accessing preimplantation genetic testing for monogenic conditions (PGT-M): a single centre retrospective cohort study of 572 IVF cycles
Source: J Assist Reprod Genet. 2025 Mar 11;42(5):1567–76. doi: 10.1007/s10815-025-03416-6 (PMC12167401; doi:10.1007/s10815-025-03416-6)
Supplement: Supplementary file 1 — Supplementary file1 List of subfertility indications categorized by female and male factors. Female subfertility indications include diminished ovarian reserve (diminished ovarian reserve, Fragile X carrier, Fragile X carrier with low AMH, low AMH), increased ovarian reserve (polycystic ovaries, polycystic ovary syndrome), ovulation disorders, factors impacting embryo implantation (endometriosis, unexplained endometriosis, uterine fibroids), unexplained subfertility (idiopathic), and other factors (chromosome mosaic, previous ectopic pregnancy). Male subfertility indications include factors impacting sperm production (azoospermia), sperm shape (oligoasthenoteratozoospermia, teratozoospermia), obstructive causes (congenital absence of the vas deferens, cystic fibrosis carrier), unexplained subfertility (idiopathic), and other factors (testicular cancer). (PDF 47 KB) [file 10815_2025_3416_MOESM1_ESM.pdf]

Title: IVF success rates in individuals accessing preimplantation genetic testing for monogenic conditions (PGT-M): a single centre retrospective cohort study of 572 IVF cycles
Journal: Journal of Assited Reproduction and Genetics

Supplementary table 1. List of subfertility indications

**Female subfertility indications**
**Diminished ovarian reserve**<br>• Diminished ovarian reserve<br>• Fragile X carrier<br>• Fragile X carrier, low AMH<br>• Low AMH
**Increased ovarian reserve**<br>• Polycystic ovaries<br>• Polycystic ovary syndrome
**Ovulation disorder**<br>• Ovulation disorders
**Impact embryo implantation**<br>• Endometriosis<br>• Endometriosis, unexplained<br>• Uterine fibroids
**Unexplained**<br>• Idiopathic
**Other**<br>• Chromosome mosaic<br>• Previous ectopic

**Male subfertility indications**
**Impacting sperm production**<br>• Azoospermia
**Impacting sperm shape**<br>• Oligoasthenoteratozoospermia<br>• Teratozoospermia
**Obstructive**<br>• Congenital abcence of the vas deferens<br>• Cystic fibrosis carrier
**Unexplained**<br>• Idiopathic
**Other**<br>• Testicular cancer
