# Supplementary material for: IVF success rates in individuals accessing preimplantation genetic testing for monogenic conditions (PGT-M): a single centre retrospective cohort study of 572 IVF cycles
Source: J Assist Reprod Genet. 2025 Mar 11;42(5):1567–76. doi: 10.1007/s10815-025-03416-6 (PMC12167401; doi:10.1007/s10815-025-03416-6)
Supplement: Supplementary file 2 — Supplementary file2 Detailed embryo testing outcomes for PGT-A and PGT-M cycles. The table presents the outcomes of 2344 embryos tested, categorized by the presence of aneuploidy, inconclusive results, mosaicism, euploid status, biopsy taken but testing not performed, result pending, and DNA amplification failure. Outcomes are further detailed by the risk for the condition of interest: high risk (876 embryos), low risk (843 embryos), carrier status (330 embryos), inconclusive due to aneuploidy in the region of interest (22 embryos), and other inconclusive results (92 embryos). Additional categories include biopsy taken but testing not performed (77 embryos), result pending (5 embryos), and DNA amplification failure (99 embryos). (PDF 47.5 KB) [file 10815_2025_3416_MOESM2_ESM.pdf]

Title: IVF success rates in individuals accessing preimplantation genetic testing for monogenic conditions (PGT-M): a single centre retrospective cohort study of 572 IVF cycles
Journal: Journal of Assited Reproduction and Genetics

Supplementary table 2. Detailed embryo testing outcomes

		**PGT-A**							
**PGT-M**	**Outcome**	**Aneuploid**	**Inconclusive**	**Mosaic**	**Euploid**	**Biopsy taken but testing not performed***	**Result pending**	**DNA amplification failure**	Total
	**High risk for condition of interest**	226	9	16	625	0	0	0	876
	**Low risk for condition of interest**	238	2	1	602	0	0	0	843
	**Carrier**	80	2	1	247	0	0	0	330
	**Inconclusive due to aneuploidy in region of interest**	22	0	0	0	0	0	0	22
	**Inconclusive**	3	85	0	4	0	0	0	92

	**Biopsy taken but testing not performed**	0	0	0	0	82	0	0	82
	**DNA amplification failure**	0	0	0	0	0	0	99	99
	**Total**	567	98	18	1478	77	5	99	2344
