# Supplementary material for: IVF success rates in individuals accessing preimplantation genetic testing for monogenic conditions (PGT-M): a single centre retrospective cohort study of 572 IVF cycles
Source: J Assist Reprod Genet. 2025 Mar 11;42(5):1567–76. doi: 10.1007/s10815-025-03416-6 (PMC12167401; doi:10.1007/s10815-025-03416-6)
Supplement: Supplementary file 3 — Supplementary file3 Demographic information categorized by inheritance pattern. The table includes maternal age (mean and median per cycle), the top three conditions per cycle, subfertility flagged, subfertility impacting ovarian reserve, subfertility impacting embryo implantation per cycle, and high FSH start dose. For autosomal recessive conditions, the mean maternal age is 34.30 and the median is 34.34, with CF, beta thalassemia, and SMA being the top conditions. For autosomal dominant conditions, the mean maternal age is 33.25 and the median is 32.84, with myotonic dystrophy, Huntington’s disease, and familial chromosomal micro deletion being the top conditions. For X-linked recessive conditions, the mean maternal age is 33.40 and the median is 32.815, with DMD, haemophilia, and Wiskott-Aldrich syndrome being the top conditions. For X-linked dominant conditions, the mean maternal age is 34.38 and the median is 34.515, with Fragile X, RP 3, and CMT XLD type 1 being the top conditions. Subfertility impacting ovarian reserve, embryo implantation, and high FSH start dose are also detailed for each inheritance pattern. (PDF 86 KB) [file 10815_2025_3416_MOESM3_ESM.pdf]

Title: IVF success rates in individuals accessing preimplantation genetic testing for monogenic conditions (PGT-M): a single centre retrospective cohort study of 572 IVF cycles
Journal: Journal of Assited Reproduction and Genetics
Supplementary table 3. Demographic information by inheritance pattern

Inheritance pattern	Maternal age mean per cycle	Maternal age median per cycle	Top 3 conditions (per cycle)	Subfertility flagged in X cases? (cycle based data)	Subfertility that impacts ovarian reserve (cycle based data)	Subfertility that impacts embryo implantation per cycle (cycle based data)	High FSH start dose
**Autosomal recessive**	34.30	34.34	1. CF<br>2. Beta thal<br>3. SMA	71 (51.4%)	7 (4.7%)	7 (4.7%)	44 (27.7%)
**Autosomal dominant**	33.25	32.84	1. Myotonic dystrophy<br>2. Huntington's disease<br>3. Familial chromosomal micro del	134 (47.9%)	5 (1.8%)	5 (1.8%)	79 (27.0%)
**X-linked recessive**	33.40	32.815	1. DMD<br>2. Haemophilia<br>3. Wiskott-Aldrich syndrome	29 (46.8%)	3 (4.8%)	3 (4.8%)	23 (35.9%)
**X-linked dominant**	34.38	34.515	1. FraX<br>2. RP 3<br>4. CMT XLD type 1	34 (63.0%)	2 (3.7%)	2 (3.7%)	20 (35.7%)
