# Supplementary material for: IVF success rates in individuals accessing preimplantation genetic testing for monogenic conditions (PGT-M): a single centre retrospective cohort study of 572 IVF cycles
Source: J Assist Reprod Genet. 2025 Mar 11;42(5):1567–76. doi: 10.1007/s10815-025-03416-6 (PMC12167401; doi:10.1007/s10815-025-03416-6)
Supplement: Supplementary file 4 — Supplementary file4 Clinical outcomes categorized by monogenic inheritance pattern. The table includes the number of cycles, embryos biopsied, embryos transferred, embryo transfer cycles, clinical pregnancies, and births (including live births and ongoing pregnancies) for each inheritance pattern: autosomal recessive, autosomal dominant, X-linked recessive, and X-linked dominant. For autosomal recessive conditions, there were 159 cycles, 673 embryos biopsied, 152 embryos transferred, 145 embryo transfer cycles, 82 clinical pregnancies, and 74 births. For autosomal dominant conditions, there were 293 cycles, 1259 embryos biopsied, 254 embryos transferred, 245 embryo transfer cycles, 130 clinical pregnancies, and 114 births. For X-linked recessive conditions, there were 64 cycles, 263 embryos biopsied, 67 embryos transferred, 62 embryo transfer cycles, 31 clinical pregnancies, and 26 births. For X-linked dominant conditions, there were 56 cycles, 149 embryos biopsied, 40 embryos transferred, 38 embryo transfer cycles, 20 clinical pregnancies, and 17 births. (PDF 36 KB) [file 10815_2025_3416_MOESM4_ESM.pdf]

Title: IVF success rates in individuals accessing preimplantation genetic testing for monogenic conditions (PGT-M): a single centre retrospective cohort study of 572 IVF cycles
Journal: Journal of Assited Reproduction and Genetics
Supplementary table 4. Clinical outcomes by monogenic inheritance pattern.

Inheritance pattern	Autosomal recessive	Autosomal Dominant	X-linked recessive	X-linked dominant
Number of cycles	159	293	64	56
Number of embryos biopsied	673	1259	263	149
Number of embryos transferred	152	254	67	40
Number of embryo transfer cycles	145	245	62	38
Number of clinical pregnancies	82	130	31	20
Births (live births and ongoing pregnancy)	74	114	26	17
