# Supplementary material for: IVF success rates in individuals accessing preimplantation genetic testing for monogenic conditions (PGT-M): a single centre retrospective cohort study of 572 IVF cycles
Source: J Assist Reprod Genet. 2025 Mar 11;42(5):1567–76. doi: 10.1007/s10815-025-03416-6 (PMC12167401; doi:10.1007/s10815-025-03416-6)
Supplement: Supplementary file 5 — Supplementary file5 Aneuploidy screening outcomes categorized by monogenic inheritance pattern. The table presents the outcomes of aneuploidy testing for embryos classified as autosomal recessive, autosomal dominant, X-linked recessive, and X-linked dominant. The outcomes include the number and percentage of embryos found to be aneuploid, with low-moderate mosaicism, euploid, inconclusive, biopsy taken but testing not performed, DNA amplification failure, and result pending. For autosomal recessive conditions, 170 embryos (25.3%) were aneuploid, 6 (0.9%) had low-moderate mosaicism, 405 (60.2%) were euploid, 27 (4.0%) were inconclusive, 29 (4.3%) had biopsy taken but testing not performed, and 36 (5.3%) experienced DNA amplification failure. For autosomal dominant conditions, 313 embryos (24.9%) were aneuploid, 8 (0.6%) had low-moderate mosaicism, 810 (64.3%) were euploid, 50 (4.0%) were inconclusive, 36 (3.0%) had biopsy taken but testing not performed, and 42 (3.3%) experienced DNA amplification failure. For X-linked recessive conditions, 52 embryos (19.8%) were aneuploid, 4 (1.5%) had low-moderate mosaicism, 164 (62.4%) were euploid, 16 (4.0%) were inconclusive, 8 (2.9%) had biopsy taken but testing not performed, and 15 (3.3%) experienced DNA amplification failure. For X-linked dominant conditions, 52 embryos (20.0%) were aneuploid, 4 (1.5%) had low-moderate mosaicism, 164 (63.1%) were euploid, 16 (6.2%) were inconclusive, 8 (3.1%) had biopsy taken but testing not performed, 15 (5.8%) experienced DNA amplification failure, and 1 (0.4%) had a result pending. (PDF 38.3 KB) [file 10815_2025_3416_MOESM5_ESM.pdf]

Title: IVF success rates in individuals accessing preimplantation genetic testing for monogenic conditions (PGT-M): a single centre retrospective cohort study of 572 IVF cycles
Journal: Journal of Assited Reproduction and Genetics
Supplementary table 5. Aneuploidy screening outcomes by monogenic inheritance pattern

Aneuploidy testing outcome	Autosomal Recessive	Autosomal Dominant	X-linked Recessive	X-linked dominant
**Aneuploid**	170 (25.3%)	315 (25.0%)	52 (19.8%)	34 (22.8%)
**Low-moderate mosaicism**	6 (0.9%)	8 (0.6%)	4 (1.5%)	0 (0%)
**Euploid**	405 (60.2%)	810 (64.3%)	164 (62.4%)	99(66.4%)
**Inconclusive**	27 (4.0%)	50 (4.0%)	16 (4.0%)	5 (3.4%)
**Biopsy taken but testing not performed**	29 (4.3%)	36 (2.9%)	12 (2.9%)	5 (3.4%)
**DNA amplification failure**	36 (5.3%)	42 (3.3%)	15 (3.3%)	6 (4.0%)
**Total**	673	1259	263	149
