# Supplementary material for: IVF success rates in individuals accessing preimplantation genetic testing for monogenic conditions (PGT-M): a single centre retrospective cohort study of 572 IVF cycles
Source: J Assist Reprod Genet. 2025 Mar 11;42(5):1567–76. doi: 10.1007/s10815-025-03416-6 (PMC12167401; doi:10.1007/s10815-025-03416-6)
Supplement: Supplementary file 6 — Supplementary file6 Monogenic screening outcomes categorized by monogenic inheritance pattern. This table provides the results of monogenic testing for embryos classified under autosomal recessive, autosomal dominant, X-linked recessive, and X-linked dominant inheritance patterns. The outcomes include the number and percentage of embryos identified as high risk for the condition of interest, low risk for the condition of interest, carrier (or affected female embryo for X-linked dominant), inconclusive due to aneuploidy in the region of interest, inconclusive, biopsy taken but testing not performed, result pending, and DNA amplification failure. For autosomal recessive conditions, 167 embryos (24.8%) were high risk, 136 (20.2%) were low risk, 270 (40.1%) were carriers, 8 (1.2%) were inconclusive due to aneuploidy in the region of interest, 27 (4.0%) were inconclusive, 29 (4.3%) had biopsy taken but testing not performed, and 36 (5.3%) experienced DNA amplification failure. For autosomal dominant conditions, 604 embryos (48.0%) were high risk, 525 (41.7%) were low risk, 9 (0.7%) were inconclusive due to aneuploidy in the region of interest, 43 (3.4%) were inconclusive, 36 (2.9%) had biopsy taken but testing not performed, and 42 (3.3%) experienced DNA amplification failure. For X-linked recessive conditions, 54 embryos (20.5%) were high risk, 120 (45.6%) were low risk, 43 (16.3%) were carriers, 2 (0.8%) were inconclusive due to aneuploidy in the region of interest, 17 (6.5%) were inconclusive, 8 (3.0%) had biopsy taken but testing not performed, 4 (1.5%) had a result pending, and 15 (5.7%) experienced DNA amplification failure. For X-linked dominant conditions, 51 embryos (34.2%) were high risk, 62 (41.6%) were low risk, 17 (11.4%) were carriers or affected female embryos, 3 (2.0%) were inconclusive due to aneuploidy in the region of interest, 5 (3.4%) were inconclusive, 4 (2.7%) had biopsy taken but testing not performed, 1 (0.7%) had a result pending, and 6 (4.0%) e [file 10815_2025_3416_MOESM6_ESM.pdf]

Title: IVF success rates in individuals accessing preimplantation genetic testing for monogenic conditions (PGT-M): a single centre retrospective cohort study of 572 IVF cycles
Journal: Journal of Assited Reproduction and Genetics
Supplementary table 6. Monogenic screening outcomes by monogenic inheritance pattern

Monogenic testing outcome	Autosomal Recessive	Autosomal Dominant	X-linked Recessive	X-linked dominant
**High risk for condition of interest**	167 (24.8%)	604 (48.0%)	54 (20.5%)	51 (34.2%)
**Low risk for condition of interest**	136 (20.2%)	525 (41.7%)	120 *45.6%)	62 (41.6%)
**Carrier (or affected female embryo for X-linked dominant)**	270 (40.1%)	0 (0%)	43 (16.3%)	17 (11.4%)
**Inconclusive due to aneuploidy in region of interest**	8 (1.2%)	9 (0.7%)	2 (0.8%)	3 (2.0%)
**Inconclusive**	27 (4.0%)	43 (3.4%)	17 (6.5%)	5 (3.4%)
**Biopsy taken but testing not performed***	29 (4.3%)	36 (2.9%)	12 (3.0%)	5 (2.7%)
**DNA amplification failure**	36 (5.3%)	42 (3.3%)	15 (5.7%)	6 (4.0%)
**Total**	673	1259	263	149
