# Supplementary material for: IVF success rates in individuals accessing preimplantation genetic testing for monogenic conditions (PGT-M): a single centre retrospective cohort study of 572 IVF cycles
Source: J Assist Reprod Genet. 2025 Mar 11;42(5):1567–76. doi: 10.1007/s10815-025-03416-6 (PMC12167401; doi:10.1007/s10815-025-03416-6)
Supplement: Supplementary file 8 — Supplementary file8 This table summarizes the GEE analysis outcomes for clinical pregnancy and live birth rates per embryo transferred, based on 421 observations from 193 groups. The number of observations per group ranges from a minimum of 1 to a maximum of 13, with an average of 2.2 observations per group. For clinical pregnancy, the Wald chi-squared statistic is 7.12 with a p-value of 0.1296, indicating no statistically significant predictors. The coefficients, standard errors, and p-values for the predictors are as follows: advanced maternal age (coefficient = 0.3691, p = 0.117), BMI high or low (coefficient = 0.0471, p = 0.824), FSH start dose 300 or above (coefficient = -0.0850, p = 0.715), and subfertility indication flagged (coefficient = -0.4797, p = 0.026). For live birth, the Wald chi-squared statistic is 5.76 with a p-value of 0.2177, also indicating no statistically significant predictors. The predictors' coefficients, standard errors, and p-values are as follows: advanced maternal age (coefficient = 0.3407, p = 0.145), BMI high or low (coefficient = 0.0726, p = 0.733), FSH start dose 300 or above (coefficient = -0.0680, p = 0.771), and subfertility indication flagged (coefficient = -0.4172, p = 0.052). (PDF 39 KB) [file 10815_2025_3416_MOESM8_ESM.pdf]

Title: IVF success rates in individuals accessing preimplantation genetic testing for monogenic conditions (PGT-M): a single centre retrospective cohort study of 572 IVF cycles
Journal: Journal of Assited Reproduction and Genetics
Supplementary 8. GEE analysis outcome per embryo transferred

Number of observations = 421

Number of groups = 193

Number of observations per group:

Min = 1, average = 2.2, max =13

	**Clinical pregnancy**		
	Wald chi2 (4) =7.12<br>Prob >chi2=0.1296		
**Predictor**	**Coefficient**	**St. error**	**p-value**
**Advance maternal age**	0.3691187	0.2353389	0.117
**BMI high or low**	0.0471098	0.2118788	0.824
**FSH start dose 300 or above**	-0.0849807	0.2330149	0.715
**Subfertility indication flagged**	-0.4797474	0.2152336	**0.026**
	**Live Birth**		
	Wald chi2 (4) =5.76<br>Prob >chi2=0.2177		
**Predictor**	Coefficient	St. error	p-value
**Advance maternal age**	0.340705	0.2336271	0.145
**BMI high or low**	0.072559	0.2123134	0.733
**FSH start dose 300 or above**	-0.0679907	0.2333212	0.771
**Subfertility indication flagged**	-0.4172361	0.2144909	0.052
