# Supplementary material for: IVF success rates in individuals accessing preimplantation genetic testing for monogenic conditions (PGT-M): a single centre retrospective cohort study of 572 IVF cycles
Source: J Assist Reprod Genet. 2025 Mar 11;42(5):1567–76. doi: 10.1007/s10815-025-03416-6 (PMC12167401; doi:10.1007/s10815-025-03416-6)
Supplement: Supplementary file 9 — Supplementary file9 (PDF 95 KB) [file 10815_2025_3416_MOESM9_ESM.pdf]

STROBE Statement—Checklist of items that should be included in reports of ***cohort studies***

	Item No	Recommendation	Page No.	Relevant text from manuscript
**Title and abstract**	1	(*a*) Indicate the study's design with a commonly used term in the title or the abstract	1	Title
		(*b*) Provide in the abstract an informative and balanced summary of what was done and what was found	3	Abstract
**Introduction**				
Background/rationale	2	Explain the scientific background and rationale for the investigation being reported	5	Introduction
Objectives	3	State specific objectives, including any prespecified hypotheses	5	Introduction
**Methods**				
Study design	4	Present key elements of study design early in the paper	5	Materials and methods
Setting	5	Describe the setting, locations, and relevant dates, including periods of recruitment, exposure, follow-up, and data collection	5	Materials and methods
Participants	6	(*a*) Give the eligibility criteria, and the sources and methods of selection of participants. Describe methods of follow-up	5	Materials and methods
		(*b*) For matched studies, give matching criteria and number of exposed and unexposed	-	-
Variables	7	Clearly define all outcomes, exposures, predictors, potential confounders, and effect modifiers. Give diagnostic criteria, if applicable	7	Materials and methods
Data sources/ measurement	8*	For each variable of interest, give sources of data and details of methods of assessment (measurement). Describe comparability of assessment methods if there is more than one group	7	Materials and methods
Bias	9	Describe any efforts to address potential sources of bias	8	Materials and methods

Study size	10	Explain how the study size was arrived at	5	Materials and methods
Quantitative variables	11	Explain how quantitative variables were handled in the analyses. If applicable, describe which groupings were chosen and why	7 and 8	Materials and methods
Statistical methods	12	(*a*) Describe all statistical methods, including those used to control for confounding	7 and 8	Materials and methods
		(*b*) Describe any methods used to examine subgroups and interactions	7	Materials and methods
		(*c*) Explain how missing data were addressed	Not applicable	
		(*d*) If applicable, explain how loss to follow-up was addressed	Not applicable	
		(*e*) Describe any sensitivity analyses	Not applicable	
**Results**				
Participants	13*	(a) Report numbers of individuals at each stage of study—eg numbers potentially eligible, examined for eligibility, confirmed eligible, included in the study, completing follow-up, and analysed	Figures 1 and 2	Results
		(b) Give reasons for non-participation at each stage	Not applicable	
		(c) Consider use of a flow diagram	Figures 1 and 2	Results
Descriptive data	14*	(a) Give characteristics of study participants (eg demographic, clinical, social) and information on exposures and potential confounders	8 and 9	Results
		(b) Indicate number of participants with missing data for each variable of interest	Not applicable	
		(c) Summarise follow-up time (eg, average and total amount)	Not applicable	

Outcome data	15*	Report numbers of outcome events or summary measures over time	8	Results
Main results	16	(*a*) Give unadjusted estimates and, if applicable, confounder-adjusted estimates and their precision (eg, 95% confidence interval). Make clear which confounders were adjusted for and why they were included	Table 2	Results
		(*b*) Report category boundaries when continuous variables were categorized	Not applicable	
		(*c*) If relevant, consider translating estimates of relative risk into absolute risk for a meaningful time period	Not applicable	
Other analyses	17	Report other analyses done—eg analyses of subgroups and interactions, and sensitivity analyses	10	Results
**Discussion**				
Key results	18	Summarise key results with reference to study objectives	11	Discussion
Limitations	19	Discuss limitations of the study, taking into account sources of potential bias or imprecision. Discuss both direction and magnitude of any potential bias	13	Discussion
Interpretation	20	Give a cautious overall interpretation of results considering objectives, limitations, multiplicity of analyses, results from similar studies, and other relevant evidence	12	Discussion
Generalisability	21	Discuss the generalisability (external validity) of the study results	12	Discussion
**Other information**				
Funding	22	Give the source of funding and the role of the funders for the present study and, if applicable, for the original study on which the present article is based	1	Title page

*Give information separately for exposed and unexposed groups.

Note: An Explanation and Elaboration article discusses each checklist item and gives methodological background and published examples of transparent reporting. The STROBE checklist is best used in conjunction with this article (freely available on the Web sites of PLoS Medicine at http://www.plosmedicine.org/, Annals of Internal Medicine at http://www.annals.org/, and Epidemiology at http://www.epidem.com/). Information on the STROBE Initiative is available at http://www.strobe-statement.org.
